# Identification of non-invasive biomarkers for predicting the radiosensitivity of nasopharyngeal carcinoma from serum microRNAs

**DOI:** 10.1038/s41598-020-61958-4

**Published:** 2020-03-20

**Authors:** Kaiguo Li, Xiaodong Zhu, Ling Li, Ruiling Ning, Zhongguo Liang, Fanyan Zeng, Fang Su, Shiting Huang, Xiaohui Yang, Song Qu

**Affiliations:** 1 Department of Radiation Oncology, Affiliated Cancer Hospital of Guangxi Medical University, Cancer Institute of Guangxi Zhuang Autonomous Region, Nanning, Guangxi 530021 P.R. China; 2 Key Laboratory of High-Incidence-Tumor Prevention & Treatment (Guangxi Medical University), Ministry of Education, Nanning, Guangxi 530021 P.R. China

**Keywords:** miRNAs, Predictive markers, Head and neck cancer

## Abstract

Serum microRNAs (miRNAs) have been reported as novel biomarkers for various diseases. But circulating biomarkers for predicting the radiosensitivity of nasopharyngeal carcinoma (NPC) have not been used in clinical practice. To screen out of differently expressed serum miRNAs from NPC patients with different radiosensitivity may be helpful for its individual therapy. NPC patients with different radiosensitivity were enrolled according to the inclusion and exclusion criteria. RNA was isolated from serum of these NPC patients before treatment. We investigated the differential miRNA expression profiles using microarray test (GSE139164), and the candidate miRNAs were validated by reverse transcription-quantitative real time polymerase chain reaction (RT-qPCR) experiments. Receiver operating characteristic (ROC) analysis has been applied to estimate the diagnostic value. In this study, 37 serum-specific miRNAs were screened out from 12 NPC patients with different radiosensitivity by microarray test. Furthermore, RT-qPCR analysis confirmed that hsa-miR-1281 and hsa-miR-6732-3p were significantly downregulated in the serum of radioresistant NPC patients (P < 0.05), which was consistent with the results of microarray test. ROC curves demonstrated that the AUC for hsa-miR-1281 was 0.750 (95% CI: 0.574–0.926, SE 87.5%, SP 57.1%). While the AUC for hsa-miR-6732-3p was 0.696 (95% CI: 0.507–0.886, SE 56.3%, SP 78.6%). These results suggested that hsa-miR-1281 and hsa-miR-6732-3p in serum might serve as potential biomarkers for predicting the radiosensitivity of NPC.

## Introduction

NPC is a squamous cell carcinoma arising from the nasopharynx epithelium^1^. Although it may be regarded as one of the rarer forms of neoplasm all over the world, it is notable for its high incidence in South China and Southeast Asia^2^. Due to the specific anatomic site and characteristic of NPC, integrated treatment pattern which radiotherapy contributed mostly was still a prevalent means at present. As is known to all, radiation resistance is one of the major obstacles to the efficacy and prognosis of NPC, and its molecular mechanism is still under study^3^. So far, no effective biomarkers have been used to predict the radiosensitivity of NPC.

Recently, with the profound study of miRNAs, new avenues for cancer diagnosis and prediction of treatment response were opened. MiRNAs are a class of naturally occurring small noncoding RNAs which are 19–25 nucleotides (19–25 nt) in length. They have recently been reported to be frequently dysregulated in various cancers^4–7^. Accumulating evidences indicate that circulating miRNAs could be stable blood-based markers for cancer diagnosis^8^. And miRNAs are involved in radioresistance by regulating cell proliferation, cell cycle, apoptosis, and tumor angiogenesis via targeting mRNA for degradation or inhibition of translation^9–12^. Although a larger number of researches have been performed to investigate the importance of miRNAs in radiosensitivity of NPC; however, no specific serum miRNAs have been applied as biomarkers to estimate the radiosensitivity of NPC in clinic as yet.

Based on miRNA profile screened out from NPC patients with different radiosensitivity, this research was conducted to investigate the correlation between serum miRNAs and radiotherapy response in NPC, and to identify biomarkers for predicting the radiosensitivity of NPC.

## Materials and Methods

### Patients and samples

Serum samples were obtained from 33 NPC patients with different radiosensitivity before anticancer therapy. All patients were confirmed as NPC by imaging tests and pathological examination in Affiliated Cancer Hospital of Guangxi Medical University from 2014 to 2019. Among them, the specimens of 12 patients with different radiosensitivity in the training cohort (including 7 radiosensitive NPC patients and 5 radioresistant NPC patients) were selected for Agilent microarray detection. And 30 patient specimens in the validation cohort (including 16 radiosensitive NPC patients and 14 radioresistant NPC patients) were used for RT-qPCR. Here, we defined the patients with <40% reduction of tumour size after radiation treatment as radioresistant NPC patients, and the patients with >60% reduction of tumour size as radiosensitive NPC patients^13,14^. It must be noted that, all patients defined as radiosensitive were treated with definitive radiation therapy only, while the patients defined as radioresistant were treated with radiotherapy with or without chemotherapy.

The study has obtained approval from the Ethics Committee of Guangxi Medical University Cancer Hospital, and it was one part of our trial study (ChiCTR-ROC-17011888). The entire experiment procedure was performed in accordance with the guidelines of Declaration of Helsinki, and written informed consents were obtained from each participant.

### Total RNA extraction from serum

About 5 mL of non-coagulant venous blood was obtained from every enrolled patient. Serum was stored at −80 °C after isolated by centrifuging at 3000 rpm for 10 min at 4 °C. Total RNA containing small RNA was extracted from serum by using the Trizol reagent (Invitrogen) and purified with mirVana miRNA Isolation Kit (Ambion, Austin, TX, USA) according to manufacter’s protocol. Briefly, chloroform was added to 200 μL of serum, which was mixed with 2× denaturing solution and incubated on ice for 5 min. After centrifuging and removing the aqueous (upper) phase, 100% ethanol was added, mixed thoroughly, pipetted onto the filter cartridge, and then centrifuged. After washing by 350 μL of wash solution three times, the filter was added with 100 μL of preheated (95 °C) nuclease-free water and centrifuged for 30 s to recover the RNA^15^.

### Microarray data analysis

Total RNA samples were analyzed by CapitalBiotech (CapitalBio Corp.) for microarray experiments. The Agilent human miRNA Array V21.0 (8 × 60 K) was applied for detection of profiling of miRNA in the serum specimens of 12 NPC patients with different radiosensitivity. Totally, the microarray covered 2,549 miRNAs from the latest miRBase database. The slide was scanned by the Agilent Microarray Scanner (Cat# G2565CA, Agilent technologies, Santa Clara, CA, US) and the Feature Exaction software 10.7 (Agilent technologies, Santa Clara, CA, US)^16^. The miRNA array data were analyzed for data summarization, normalization and quality control by using the GeneSpring software V13 (Agilent). The default 90th percentile normalization method were performed for date preprocessing. All steps were performed were according to the recommended protocol. To screen out the candidate miRNAs, threshold values of ≥2 and ≤−2 fold change and a Benjamini-Hochberg corrected p vlaue of 0.05 were used. The data was Log2 transformed and median centered by genes using the Adjust Data function of CLUSTER 3.0 software. Then we further analyzed the data with hierarchical clustering with average linkage. Finally, we performed tree visualization by using Multiple Experiment Viewer (MeV version 4_9_0).

### Primer design and qPCR validation

Total RNAs were extracted from 30 NPC serum specimens by using miRcute miRNA Isolation Kit (No. DP503, TIANGEN Biotech, Beijing, China), and were reversely transcribed into cDNA by miRcute Plus miRNA First-Strand cDNA Synthesis Kit (No. KR211, TIANGEN Biotech, Beijing, China) according to manufacter’s protocol. During this procedure, the 3′ terminal of miRNAs was polyadenylated and converted into cDNA by reverse transcriptase using oligo-dT and universal primers. The forward primers of miRNAs were ordered from Sangon Biotech (Shanghai) Co Ltd and TIANGEN Biotech for qPCR and all forward primer sequences were shown in Table 1. qPCR was performed to validate expression level of selected miRNAs by usage of miRcute Plus miRNA qPCR Detection Kit (SYBR Green) (No. FP411, TIANGEN Biotech, Beijing, China). We selected hsa-U6 as the internal control. All experiments were carried out in triplicate following the manufacturer’s instructions^17^.

**Table 1:** Specific forward primers for candidate miRNAs.

miRNA	Forward Primers	Corporation
hsa-miR-1281	TCGCCTCCTCCTCTCCC	Sangon Biotech
hsa-miR-6732-3p	TAACCCTGTCCTCTCCCTCC	Sangon Biotech
hsa-miR-6865-3p	ACACCCTCTTTCCCTACCG	Sangon Biotech
hsa-miR-1825	No. CD201-0078	TIANGEN Biotech

### Statistical analysis

We compared the clinical characteristics among groups using the Fisher’s exact test for qualitative data, and t-test for quantitative data. A Benjamini Hochberg adjusted p < 0.05 was deemed to be statistically significant. To analyze the qRT-PCR data, we calculated the relative expression levels of each candidate miRNA by using the 2^−ΔCT^ method (ΔCT = CTmiR − CTreference). The 2^−ΔCT^ dataset was then Log10-transformed when necessary^18,19^. Statistical analysis was executed using a t-test. The qPCR data was presented using GraphPad Prism 6.0. The AUC (area under ROC curve) used to assess the diagnostic power of the predictors was determined using SPSS 22.0 statistical software (SPSS, Chicago, IL, USA). Risk score analysis including ROC and AUC were performed to estimate diagnostic value of the candidate miRNAs in NPC. All statistical calculations were considered to have significantly statistical differences when P < 0.05.

### Ethical approval

All procedures performed in studies involving human participants were in accordance with the ethical standards of the institutional and/or national research committee and with the 1964 Helsinki declaration and its later amendments or comparable ethical standards.

### Informed consent

Informed consent was obtained from all individual participants included in the study.

## Results

### Patient characteristics

The characteristics of NPC patients are listed in Table 2 and S1. The median age of all the NPC patients was 47.79 years (range, 24 ~ 70 years); 28 cases (84.85%) were 60 years or younger. Men were more than women (ratio, 2:1). It was found that patients with stage III/IV accounted for 51.52% of cases. 20 of the patiens (60.61%) treated with radiotherapy only. In the traing cohort, there was no baseline difference in patient characteristics between two groups in terms of sex, age, and stage (Fisher’s exact test, P > 0.05, Table 2).

**Table 2:** The characteristics of NPC samples.

Characteristic	The training cohort			The validation cohort		
	Radiosensitive n = 7 (%)	Radioresistant n = 5 (%)	*P value*	Radiosensitive n = 16 (%)	Radioresistant n = 14 (%)	*P value*
Sex			0.576			0.709
male	4 (33.33%)	4 (33.33%)		10 (62.50%)	10 (71.43%)	
female	3 (25.00%)	1 (8.33%)		6 (37.50%)	4 (28.57%)	
Age(y)			1.000			1.000
<60	5 (41.67%)	4 (33.33%)		13 (81.25%)	12 (85.71%)	
≥60	2 (16.67%)	1 (8.33%)		3 (18.75%)	2 (14.29%)	
Stage^a^			0.293			0.004
I/II	4 (33.33%)	1 (8.33%)		11 (68.75%)	2 (14.29%)	
III/IV	3 (25.00%)	4 (33.33%)		5 (31.25%)	12 (85.71%)	

^a^Classification according to the American Joint Committee on Cancer, 7th edition.

### Profiling of differentially expressed miRNAs

**Figure 1:**
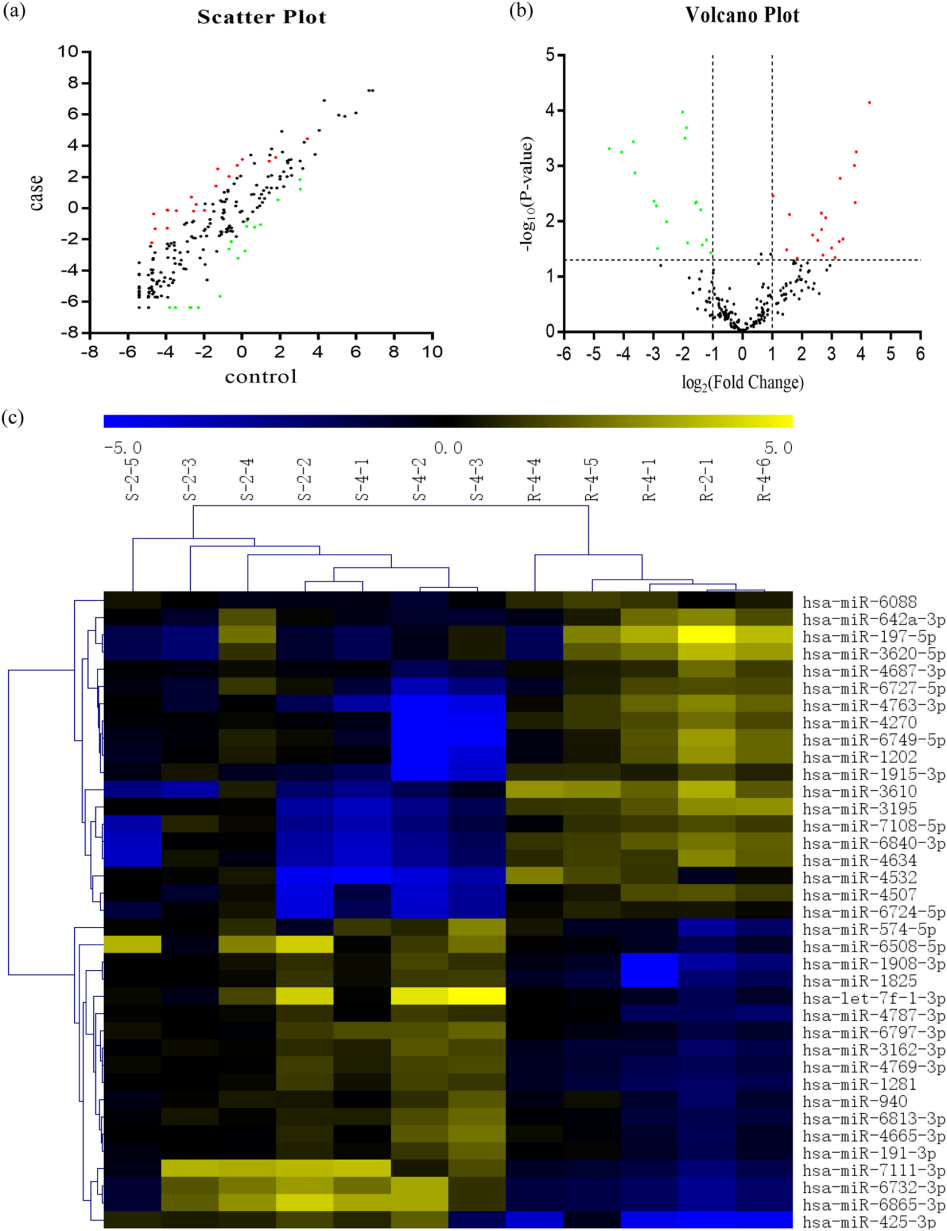
Profiling of the microarray data: (**a**) Scatter plot is performed to visualize the expression variation of differentially expressed miRNAs between NPC with different radiosensitivity. The values of X and Y axis are log2 scaled intensity values of miRNA microarray probes. Red color implies miRNAs in higher expression level and green one signifies miRNAs in lower expression level. (**b**) Volcano plot is presented to show significantly differentially expressed miRNAs (P < 0.05). Fold change values are log2 transformed as the X axis while the log10 transformation of P values are set in the Y axis. (**c**) Hierarchical clustering is conducted to show the expression patterns of differentially expressed miRNAs in 12 samples.

After normalization of raw data by GeneSpring software, scatter plot was applied to represent the expression variation of miRNAs. The findings showed that fold change of quite a few of miRNAs were more than two times between the two groups (Fig. 1a). Further, volcano plot showed miRNAs with significantly statistical differences (fold-change ≥2 or ≤0.5 and P < 0.05; Fig. 1b). Hierarchical clustering was then applied to figure out the expression pattern of these differentially expressed miRNAs (Fig. 1c). On basis of screening criteria (fold-change ≥2 or ≤0.5 and P < 0.05), only 19 miRNAs in higher expression level (including hsa-miR-4507, hsa-miR-6749-5p, hsa-miR-108-5p, hsa-miR-4763-3p, hsa-miR-6727-5p, hsa-miR-197-5p, hsa-miR-4270, hsa-miR-642a-3p, hsa-miR-3610, hsa-miR-4687-3p, hsa-miR-3620-5p, hsa-miR-4532, hsa-miR-1202, hsa-miR-3195, hsa-miR-6840-3p, hsa-miR-4634, hsa-miR-6088, hsa-miR-1915-3p and hsa-miR-6724-5p) and 18 miRNAs in lower expression level (including hsa-miR-3162-3p, hsa-miR-6813-3p, hsa-miR-1908-3p, hsa-miR-6732-3p, hsa-miR-574-5p, hsa-miR-7111-3p, hsa-miR-1825, hsa-miR-4787-3p, hsa-miR-6797-3p, hsa-miR-4769-3p, hsa-miR-6508-5p, hsa-let-7f-1-3p, hsa-miR-6865-3p, hsa-miR-1281, hsa-miR-4665-3p, hsa-miR-940, hsa-miR-191-3p, hsa-miR-425-3p) were identified to have strong significantly statistical differences between NPC patients with different radiosensitivity (Table 3).

**Table 3:** Summary of differentially expressed miRNAs in NPC with different radiosensitivity.

	miRNA	Expression level	Fold change	*P* value
1	hsa-miR-4507	↑	6.326725	0.014001
2	hsa-miR-6749-5p	↑	8.685177	0.045365
3	hsa-miR-108-5p	↑	6.294073	0.007129
4	hsa-miR-4763-3p	↑	13.91462	0.004562
5	hsa-miR-6727-5p	↑	3.581773	0.046507
6	hsa-miR-197-5p	↑	9.546075	0.023115
7	hsa-miR-4270	↑	8.022163	0.030378
8	hsa-miR-642a-3p	↑	2.815056	0.032839
9	hsa-miR-3610	↑	19.38883	7.1E-05
10	hsa-miR-4687-3p	↑	3.003508	0.007564
11	hsa-miR-3620-5p	↑	5.808329	0.022097
12	hsa-miR-4532	↑	10.41947	0.020743
13	hsa-miR-1202	↑	6.554704	0.04072
14	hsa-miR-3195	↑	9.770501	0.001664
15	hsa-miR-6840-3p	↑	14.2069	0.000556
16	hsa-miR-4634	↑	13.63916	0.000976
17	hsa-miR-6088	↑	2.037823	0.003464
18	hsa-miR-1915-3p	↑	6.979248	0.00864
19	hsa-miR-6724-5p	↑	5.135607	0.017725
20	hsa-miR-3162-3p	↓	3.815279	0.000311
21	hsa-miR-6813-3p	↓	2.927375	0.004488
22	hsa-miR-1908-3p	↓	7.889484	0.004336
23	hsa-miR-6732-3p	↓	12.70422	0.000364
24	hsa-miR-574-5p	↓	3.590187	0.024629
25	hsa-miR-7111-3p	↓	12.27265	0.001328
26	hsa-miR-1825	↓	7.464135	0.005255
27	hsa-miR-4787-3p	↓	2.998474	0.004655
28	hsa-miR-6797-3p	↓	2.647008	0.006168
29	hsa-miR-4769-3p	↓	3.692053	0.000202
30	hsa-miR-6508-5p	↓	5.863555	0.010158
31	hsa-let-7f-1-3p	↓	7.234791	0.030837
32	hsa-miR-6865-3p	↓	16.71123	0.000559
33	hsa-miR-1281	↓	4.036213	0.000106
34	hsa-miR-4665-3p	↓	2.546382	0.02678
35	hsa-miR-940	↓	2.31099	0.0217
36	hsa-miR-191-3p	↓	2.101149	0.037093
37	hsa-miR-425-3p	↓	22.41298	0.000487

↑: upregulated; ↓: downregulated.

### Validation of differentially expressed miRNAs

**Figure 2:**
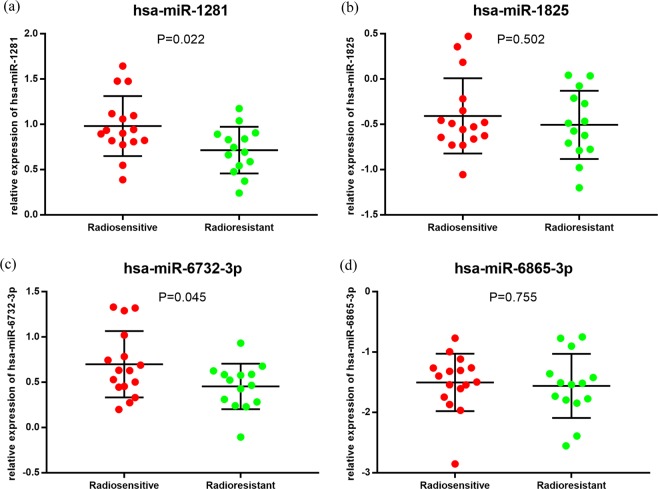
Validation of candidate miRNAs in 30 NPC patients with different radiosensitivity by qPCR. Relative expression of 4 candidate miRNAs, including hsa-miR-1281 (**a**), hsa-miR-1825 (**b**), hsa-miR-6732-3p (**c**) and hsa-miR-6865-3p (**d**). Data are presented as the mean with standard deviation (SD).

For economic and other reasons, we selected 12 most significant differentially expressed miRNAs from these 37 miRNAs^20,21^ (P ≤ 0.006, fold change ≥4), including hsa-miR-425-3p, hsa-miR-3610, hsa-miR-1281, hsa-miR-6732-3p, hsa-miR-6840-3p, hsa-miR-6865-3p, hsa-miR-4634, hsa-miR-7111-3p, hsa-miR-3195, hsa-miR-1908-3p, hsa-miR-4763-3p, hsa-miR-1825. We designed the primers for these 12 miRNAs. And four miRNAs which have the best specify primers (hsa-miR-1281, hsa-miR-1825, hsa-miR-6732-3p and hsa-miR-6865-3p) were selected to confirm the results of microarray analysis by qPCR (Fig. 2 and Table 4). In the validation phase, two miRNAs (including hsa-miR-1281 and hsa-miR-6732-3p) were identified as significantly differentially expressed miRNAs (t-test, P < 0.05: Fig. 2a,c), while the remaining two miRNAs (including hsa-miR-1825 and hsa-miR-6865-3p) having no statistical differences (t-test, P > 0.05: Fig. 2b,d).

**Table 4:** Relative expression of 4 candidate miRNAs (x ± s).

miRNA	radiosensitive (n = 16)	radioresistant (n = 14)	*P value*
hsa-miR-1281	1.00 ± 0.36	0.71 ± 0.26	0.015
hsa-miR-1825	−0.36 ± 0.45	−0.51 ± 0.38	0.366
hsa-miR-6732-3p	0.72 ± 0.38	0.45 ± 0.25	0.035
hsa-miR-6865-3p	−1.40 ± 0.64	−1.56 ± 0.53	0.454

### Prediction of candidate miRNAs target genes

Putative target genes of *candidate* miRNAs were predicted by miRWalk2.0 (http://zmf.umm.uni-heidelberg.de/apps/zmf/mirwalk2/miRretsys-self.html) which including 11 databases (miRWalk, Microt4, miRanda, mirbridge, miRDB, miRMap, miRNAMap, Pictar2, PITA, RNA22, RNAhybrid, and Targetscan). The miRNA target genes recorded by ≥8 databases (ST3GAL2, KIAA1755, PRELP, SCN4B, SLC6A4, SOX4, SRF, VAMP1, DGCR14, N4BP3, BRD4, ZDHHC8, CYP26B1, SMCO4, UNC119B, C1QL4, CCND1, CRX, DLAT, MED22, ARHGAP31 and PLEKHA2) were selected and subjected to further investigation.

### Confirmation of specificity and sensitivity of candidate miRNAs

**Figure 3:**
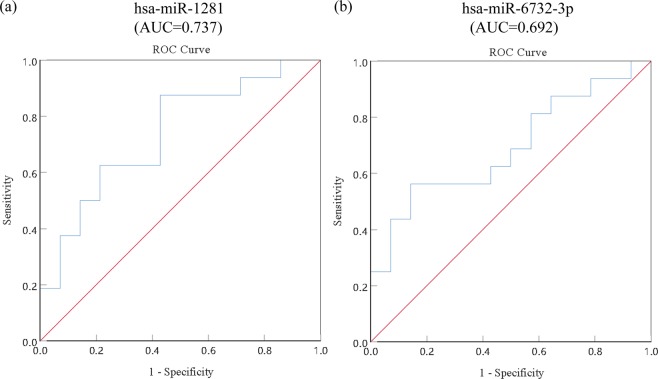
Analysis of diagnostic capability of 2 candidate miRNAs in NPC: ROC curve analysis of the 2 candidate miRNAs, including hsa-miR-1281 (**a**) and hsa-miR-6732-3p (**b**).

Through RT-qPCR test, we found that the relative expression levels of hsa-miR-1281 and hsa-miR-6732-3p in serum of the radioresistant NPC patients were significantly different from those of the radiosensitive NPC patients (P < 0.05, Table 4), which was consistent with the results of microarray test. AUC and ROC were then used to verify that if they are both suitable to serve as accurate biomarkers for estimating the radiosensitivity of NPC. The results demonstrated that the AUC of hsa-miR-1281 was 0.750 (95% CI: 0.574-0.926, P = 0.020, Fig. 3 and Table 5), and the sensitivity and specificity were 87.5% and 57.1%, respectively (Table 5). While the AUC of hsa-miR-6732-3p was 0.696 (95% CI: 0.507–0.886, P = 0.067, Fig. 3 and Table 5), and the sensitivity and specificity were 56.3% and 85.7%, respectively (Table 5).

**Table 5:** Illustration of area under the curve of 2 candidate miRNAs.

miRNA	AUC	Std. Error	*P value*	95%CI	Cut-off point	Sensitivity	Specificity
hsa-miR-1281	0.750	0.090	0.020	0.574–0.926	0.760	87.5%	57.1%
hsa-miR-6732-3p	0.696	0.097	0.067	0.507–0.886	0.607	56.3%	78.6%

AUC, areas under the receiver-operating-characteristic curve; Std. Error, standard error.

## Discussion

Up to now, radioresistance is a main bottleneck in the treatment of NPC worldwide. A large number of studies on miRNAs associated with NPC emerged in recent years, and many miRNAs were demonstrated to play important roles in regulating the radiosensitivity of NPC. Tian Y *et al*.^22^ found that overexpression of miR-483-5p decreased the radiosensitivity of NPC cells *in vitro* and *in vivo* by decreasing radiation-induced apoptosis and DNA damage, and by increasing NPC cell colony formation, via targeting death-associated protein kinase 1 (DAPK1). It was also reported that miR-29-3p could improved radiosensitivity of NPC cells by targeting COL1A1 3′-UTR^23^. Additionally, g’sudy showed that miR-206 enhanced NPC radiosensitivity by directly targeting IGF1 and inhibiting the PI3K/AKT pathway^24^. However, no effective biomarker for predicting the radiosensitivity of NPC was applied in clinic yet.

As far as we know, few studies on miRNA expression profiles associated with NPC radioresistance were reported until now. Xin-Hui Li *et al*.^25^ identified 15 differentially expressed miRNAs in the radioresistant CNE2-IR cells as compared with the radiosensitive CNE2 cells using microarray. Among them, four miRNAs (miR-762, miR-1202, miR-193b and let-7e) were upregulated, and eleven miRNAs (miR-203, miR-23a, miR-24, miR-30a, miR-545, miR-660, miR-4291, miR-183*, miR-130a, miR-31* and miR-30a*) were downregulated in the radioresistant CNE2-IR cells. Guo Li *et al*.^26^ screened out of 50 known and 9 novel miRNAs associated with radioresistance in NPC using next generation deep sequencing. And RT-qPCR assays confirmed 3 up-regulated miRNAs (miR-34c-5p, miR-371a-5p and miR-1323), 3 down-regulated miRNAs (miR-93-3p, miR-324-3p and miR-4501) and 2 novel miRNAs. Their studies provided an overview of miRNA expression profile, and further pointed out the directions for the mechanisms of miRNAs in NPC radioresistance. However, both of the above studies specimens are from NPC cell lines cultured *in vitro*. And the PCR validation specimens are mostly from NPC tissues or cells cultured *in vitro*. There are so many differences between the microenvironment of NPC cells cultured *in vitro* and NPC cells existing *in vivo*. And NPC tissue acquisition is an invasive procedure with high risk. Recently, a study^27^ demonstrated that miRNAs existed in the serum of humans. The expression of miRNAs in serum are consistent, reproducible, and stable among individuals of the same species. Therefore, it may be a better choice to select the serum miRNAs to serve as potential biomarkers. To our knowledge, few studies have been reported on serum miRNAs to serve as biomarkes for predicting the radiosensitvity of NPC until now.

In our study, we used Agilent microarray to screen the differential expression profiles of miRNAs in the serums of 12 NPC patients with different radiosensitivity. Based on the criteria set as P < 0.05 and fold change ≥2 or ≤−2, the investigation screened out that 37 differentially expressed miRNAs have significantly statistical differences. Then, four candidate miRNAs (including hsa-miR-1281, hsa-miR-1825, hsa-miR-6732-3p and hsa-miR-6865-3p) were selected to be validated by RT-qPCR in 30 patients with NPC. Among them, hsa-miR-1281 and hsa-miR-6732-3p were presented to be underlying biomarkers for estimating the radiosensitivity of NPC.

There were no studies concerning the two differentially expressed miRNAs (including hsa-miR-1281 and hsa-miR-6732-3p) in NPC till now. Abnormal expression of hsa-miR-1281 has been reported in other cancers, such as malignant pleural mesothelioma (MPM)^28^ and adrenocortical tumors^29^. A previous study showed that hsa-miR-1281 is up-regulated in MPM, and the finding suggested that hsa-miR-1281 was a potential biomarker for the diagnosis of MPM^28^. In our study, the hsa-miR-1281 has been proven to have the best ROC diagnostic value with an AUC value of 0.750, and it can be served as a promising biomarker in predicting the radiosensitivity of NPC. Otherwise, hsa-miR-6732-3p has a ROC diagnostic value with an AUC value of 0.696. However, hsa-miR-6732-3p has never been reported to associate with any cancer. And this is the first time, to our knowledge, that we present its roles as a biomarker for predicting the radiosensitivity of NPC.

In summary, our study applied the Agilent microarray to determine expression profiles of the miRNAs in serums of NPC with different radiosensitivity. Our results found two new miRNAs (hsa-miR-1281 and hsa-miR-6732-3p) that have the potential to be biomarkers for predicting the radiosensitivity of NPC, and they could be therapeutic targets for improving the radiosensitivity of NPC. However, there exist some limitions in our study. Firstly, we just find that the abnormal expressions of miRNAs in the serum of NPC patients are related to the radiosensitivity of NPC, but we haven’t yet figured out the causal relationship between them. Secondly, for various reasons, we have not validated all the differentially expressed miRNAs screened out from the microarray test. Otherwise, our findings in this study need to be confirmed in future research using multicenter institutions and larger populations. Actually, more researches using microarray profiling of miRNAs may be helpful to pave the most efficient and consolidated way for the molecular mechanisms in the radioresistance of NPC in the future.

## Conclusions

Our study maybe the first to identify 37 differentially expressed circulating miRNAs in the serum of NPC patients with different radiosensitivity. Among the four validated miRNAs (including hsa-miR-1281, hsa-miR-1825, hsa-miR-6732-3p and hsa-miR-6865-3p), hsa-miR-1281 and hsa-miR-6732-3p in serum might serve as potential biomarkers for predicting the radiosensitivity of NPC.

## Supplementary information

Supplementary information.

## Data Availability

The microarray data have been deposited in NCBI’s Gene Expression Omnibus and are accessible through GEO Series accession number GSE139164. The datasets generated during and/or analyzed during the current study are available from the corresponding author on reasonable request, or included in the published article and the supplemental material.
