# Supplementary material for: Identification of non-invasive biomarkers for predicting the radiosensitivity of nasopharyngeal carcinoma from serum microRNAs
Source: Sci Rep. 2020 Mar 20;10:5161. doi: 10.1038/s41598-020-61958-4 (PMC7083955; doi:10.1038/s41598-020-61958-4)
Supplement: Supplementary file 1 — Supplementary information. [file 41598_2020_61958_MOESM1_ESM.pdf]

Identification of non-invasive biomarkers for predicting the radiosensitivity of nasopharyngeal carcinoma from serum microRNAs

Kaiguo Li [1], Xiaodong Zhu [1,2], Ling Li [1], Ruiling Ning [1], Zhongguo Liang [1], Fanyan Zeng [1], Fang Su [1], Shiting Huang [1], Xiaohui Yang [1] , Song Qu [1,2,*]

Table-S1. Clinical data of the study population

Patient	Sex	Age(y)	Stage	Therapeutic regime	Radiosensitivity
S-1-1	male	24	I	radiotherapy	radiosensitive
S-1-2	female	44	I	radiotherapy	radiosensitive
S-2-1	male	32	II	radiotherapy	radiosensitive
S-2-2	male	49	II	radiotherapy	radiosensitive
S-2-3	female	47	II	radiotherapy	radiosensitive
S-2-4	male	34	II	radiotherapy	radiosensitive
S-2-5	male	60	II	radiotherapy	radiosensitive
S-2-6	female	52	II	radiotherapy	radiosensitive
S-2-7	male	44	II	radiotherapy	radiosensitive
S-2-8	male	56	II	radiotherapy	radiosensitive
S-2-9	male	51	II	radiotherapy	radiosensitive
S-2-10	female	49	II	radiotherapy	radiosensitive
S-2-11	female	53	II	radiotherapy	radiosensitive
S-2-12	male	56	II	radiotherapy	radiosensitive
S-3-1	male	68	III	radiotherapy	radiosensitive
S-3-2	male	65	III	radiotherapy	radiosensitive
S-4-1	male	41	IV	radiotherapy	radiosensitive
S-4-2	female	36	IV	radiotherapy	radiosensitive
S-4-3	female	64	IV	radiotherapy	radiosensitive
R-2-1	male	28	II	radiotherapy	radioresistant
R-2-2	female	31	II	concurrent chemoradiotherapy	radioresistant
R-3-1	male	37	III	radiotherapy	radioresistant
R-3-2	female	70	III	radiotherapy	radioresistant
R-4-1	male	59	IV	concurrent chemoradiotherapy	radioresistant
R-4-2	male	30	IV	concurrent chemoradiotherapy	radioresistant
R-4-3	female	50	IV	concurrent chemoradiotherapy	radioresistant

R-4-4	female	66	IV	radiotherapy	radioresistant
R-4-5	male	44	IV	induction chemotherapy + concurrent radiochemotherapy	radioresistant
R-4-6	male	50	IV	concurrent chemoradiotherapy	radioresistant
R-4-7	male	45	IV	induction chemotherapy + concurrent radiochemotherapy	radioresistant
R-4-8	male	59	IV	induction chemotherapy + concurrent radiochemotherapy	radioresistant
R-4-9	male	47	IV	concurrent chemoradiotherapy	radioresistant
R-4-10	male	36	IV	concurrent chemoradiotherapy	radioresistant

Table-S2-a. Chi-Square Tests of sex for the training cohort

	Value	df	Asymptotic Significance (2-sided)	Exact Sig. (2-sided)	Exact Sig. (1-sided)
Pearson Chi-Square	.686[a]	1	.408		
Continuity Correction[b]	.043	1	.836		
Likelihood Ratio	.712	1	.399		
**Fisher's Exact Test**				**.576**	**.424**
Linear-by-Linear Association	.629	1	.428		
N of Valid Cases	12				

a. 4 cells (100.0%) have expected count less than 5. The minimum expected count is 1.67.

b. Computed only for a 2x2 table

Table-S2-b. Chi-Square Tests of age for the training cohort

	Value	df	Asymptotic Significance (2-sided)	Exact Sig. (2-sided)	Exact Sig. (1-sided)
Pearson Chi-Square	.114[a]	1	.735		
Continuity Correction[b]	.000	1	1.000		
Likelihood Ratio	.116	1	.733		
**Fisher's Exact Test**				**1.000**	**.636**
Linear-by-Linear Association	.105	1	.746		
N of Valid Cases	12				

a. 3 cells (75.0%) have expected count less than 5. The minimum expected count is 1.25.

b. Computed only for a 2x2 table

Table-S2-c. Chi-Square Tests of stage for the training cohort

	Value	df	Asymptotic Significance (2-sided)	Exact Sig. (2-sided)	Exact Sig. (1-sided)
Pearson Chi-Square	1.656[a]	1	.198		
Continuity Correction[b]	.480	1	.488		
Likelihood Ratio	1.736	1	.188		
**Fisher's Exact Test**				**.293**	**.247**
Linear-by-Linear Association	1.518	1	.218		
N of Valid Cases	12				

a. 4 cells (100.0%) have expected count less than 5. The minimum expected count is 2.08.

b. Computed only for a 2x2 table

Table-S2-d. Chi-Square Tests of sex for the validation cohort

	Value	df	Asymptotic Significance (2-sided)	Exact Sig. (2-sided)	Exact Sig. (1-sided)
Pearson Chi-Square	.268[a]	1	.605		
Continuity Correction[b]	.017	1	.897		
Likelihood Ratio	.269	1	.604		
**Fisher's Exact Test**				**.709**	**.450**
Linear-by-Linear Association	.259	1	.611		
N of Valid Cases	30				

a. 1 cells (25.0%) have expected count less than 5. The minimum expected count is 4.67.

b. Computed only for a 2x2 table

Table-S2-e. Chi-Square Tests of age for the validation cohort

	Value	df	Asymptotic Significance (2-sided)	Exact Sig. (2-sided)	Exact Sig. (1-sided)
Pearson Chi-Square	.107[a]	1	.743		
Continuity Correction[b]	.000	1	1.000		
Likelihood Ratio	.108	1	.743		
**Fisher's Exact Test**				**1.000**	**.567**
Linear-by-Linear Association	.104	1	.748		
N of Valid Cases	30				

a. 2 cells (50.0%) have expected count less than 5. The minimum expected count is 2.33.

b. Computed only for a 2x2 table

Table-S2-f. Chi-Square Tests of stage for the validation cohort

	Value	df	Asymptotic Significance (2-sided)	Exact Sig. (2-sided)	Exact Sig. (1-sided)
Pearson Chi-Square	9.020[a]	1	.003		
Continuity Correction[b]	6.938	1	.008		
Likelihood Ratio	9.696	1	.002		
**Fisher's Exact Test**				**.004**	**.004**
Linear-by-Linear Association	8.719	1	.003		
N of Valid Cases	30				

a. 0 cells (0.0%) have expected count less than 5. The minimum expected count is 6.07.

b. Computed only for a 2x2 table

Table-S3. Summary of selected 12 miRNAs in NPC with different radiosensitivity

miRNA	Expression level	Fold change	*P* value	Mature sequence	Forward primer
hsa-miR-3610	↑	19.38883	7.10E-05	GAAUCGGAAAGGAGGCGCCG	GAATCGGAAAGGAGGCGCC
**hsa-miR-1281**	↓	**4.036213**	**0.000106**	**UCGCCUCCUCCUCUCCC**	**TCGCCTCCTCCTCTCCC**
**hsa-miR-6732-3p**	↓	**12.70422**	**0.000364**	**UAACCCUGUCCUCUCCCUCCCAG**	**TAACCCTGTCCTCTCCCTCC**
hsa-miR-425-3p	↓	22.41298	0.000487	AUCGGGAAUGUCGUGUCCGCCC	ATCGGGAATGTCGTGTCCG
hsa-miR-6840-3p	↑	14.2069	0.000556	GCCCAGGACUUUGUGCGGGGUG	GCCCAGGACTTTGTGCGG
**hsa-miR-6865-3p**	↓	**16.71123**	**0.000559**	**ACACCCUCUUUCCCUACCGCC**	**ACACCCTCTTTCCCTACCG**
hsa-miR-4634	↑	13.63916	0.000976	CGGCGCGACCGGCCCGGGG	attaCGGCGCGACCGGCCCGG
hsa-miR-7111-3p	↓	12.27265	0.001328	AUCCUCUCUUCCCUCCUCCCAG	ATCCTCTCTTCCCTCCTCCCAG
hsa-miR-3195	↑	9.770501	0.001664	CGCGCCGGGCCCGGGUU	atatttCGCGCCGGGCCCGGGTT
hsa-miR-1908-3p	↓	7.889484	0.004336	CCGGCCGCCGGCUCCGCCCCG	atattCCGGCCGCCGGCTCCGC
hsa-miR-4763-3p	↑	13.91462	0.004562	AGGCAGGGGCUGGUGCUGGGCGGG	atAGGCAGGGGCTGGTGCT
**hsa-miR-1825**	↓	**7.464135**	**0.005255**	**UCCAGUGCCCUCCUCUCC**	**No. CD201-0078（TIANGEN Biotech）**

↑: upregulated; ↓: downregulated.

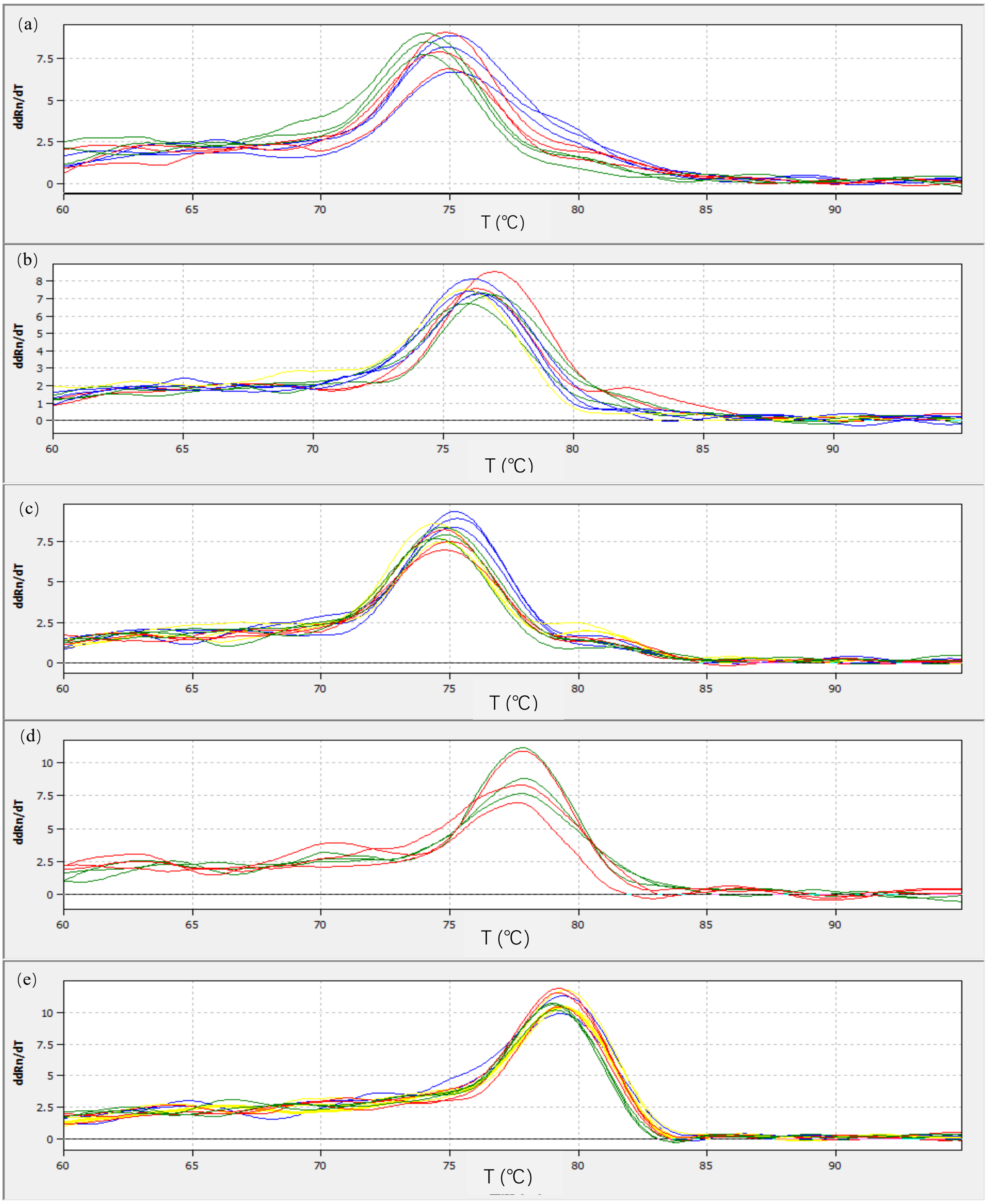

Figure-S1: Melting curves of candidate miRNAs (a.hsa-miR-1281; b.hsa-miR-1825; c.hsa-miR-6732-3p; d.hsa-miR-6865-3p; e.U6)

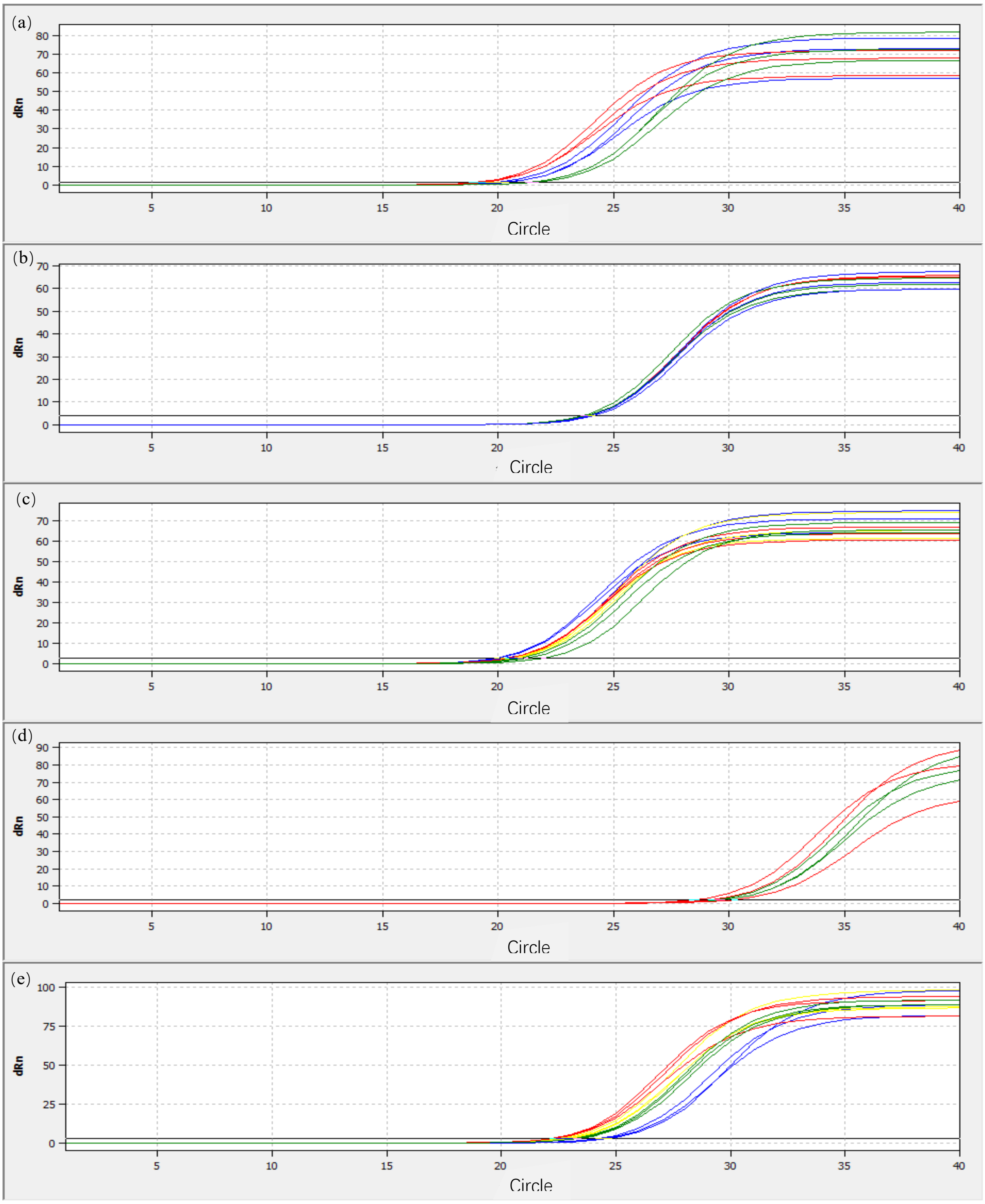

Figure-S2: Amplification curves of candidate miRNAs (a.hsa-miR-1281; b.hsa-miR-1825; c.hsa-miR-6732-3p; d.hsa-miR-6865-3p; e.U6)

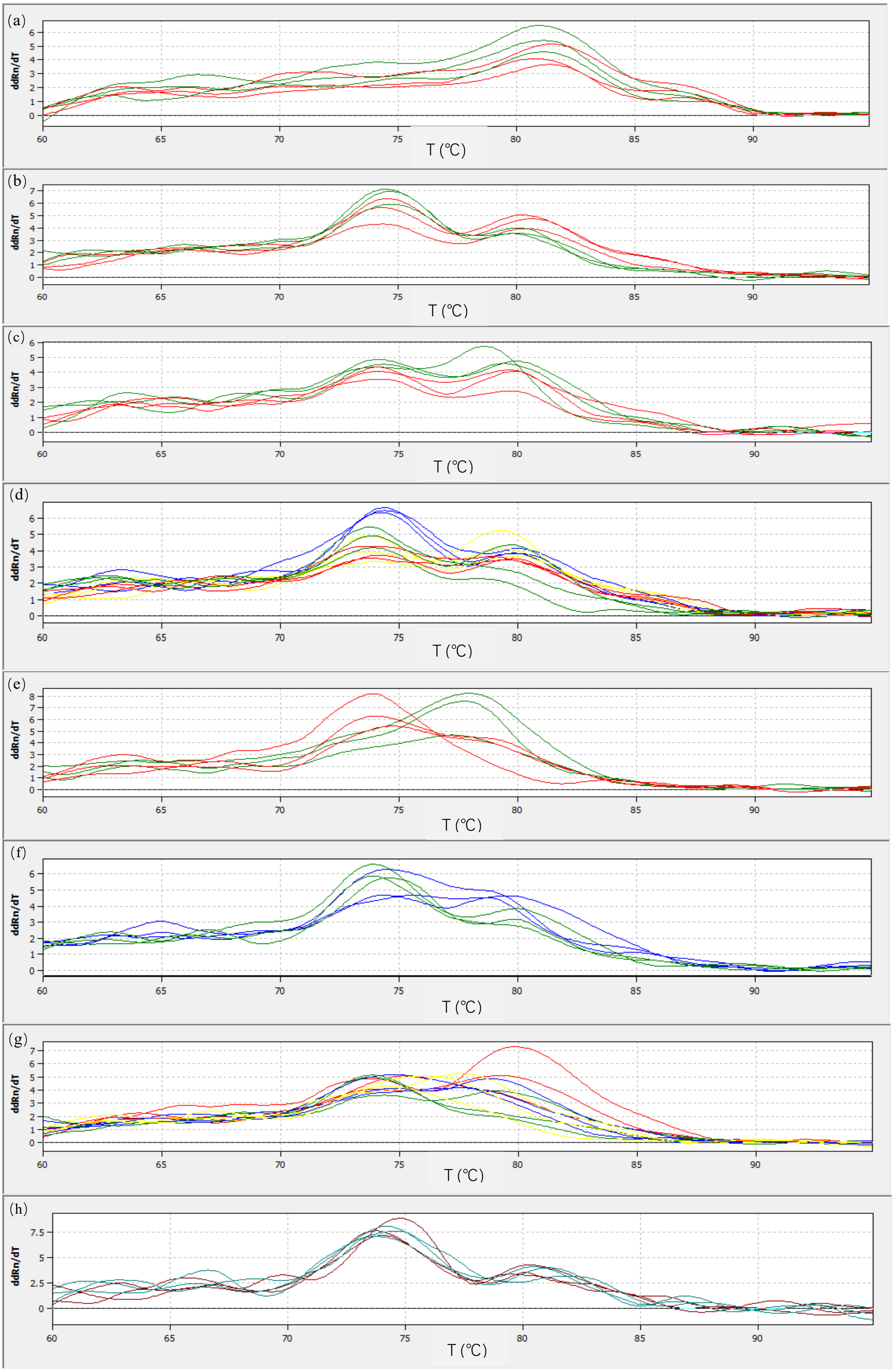
(a)
(b)
(c)
(d)
(e)
(f)
(g)
(h)
ddRn/dT
T (℃)

Figure-S3: Some melting curves of other selected miRNAs whose products were unacceptable (a. hsa-miR-4634; b. hsa-miR-425-3p; c. hsa-miR-3610; d. hsa-miR-6840-3p; e. hsa-miR-7111-3p; f. hsa-miR-3195; g. hsa-miR-1908-3p; h. hsa-miR-4763-3p)
